# Whither Sir William?

**DOI:** 10.5195/jmla.2017.225

**Published:** 2017-04

**Authors:** Stephen J. Greenberg

**Figure:**
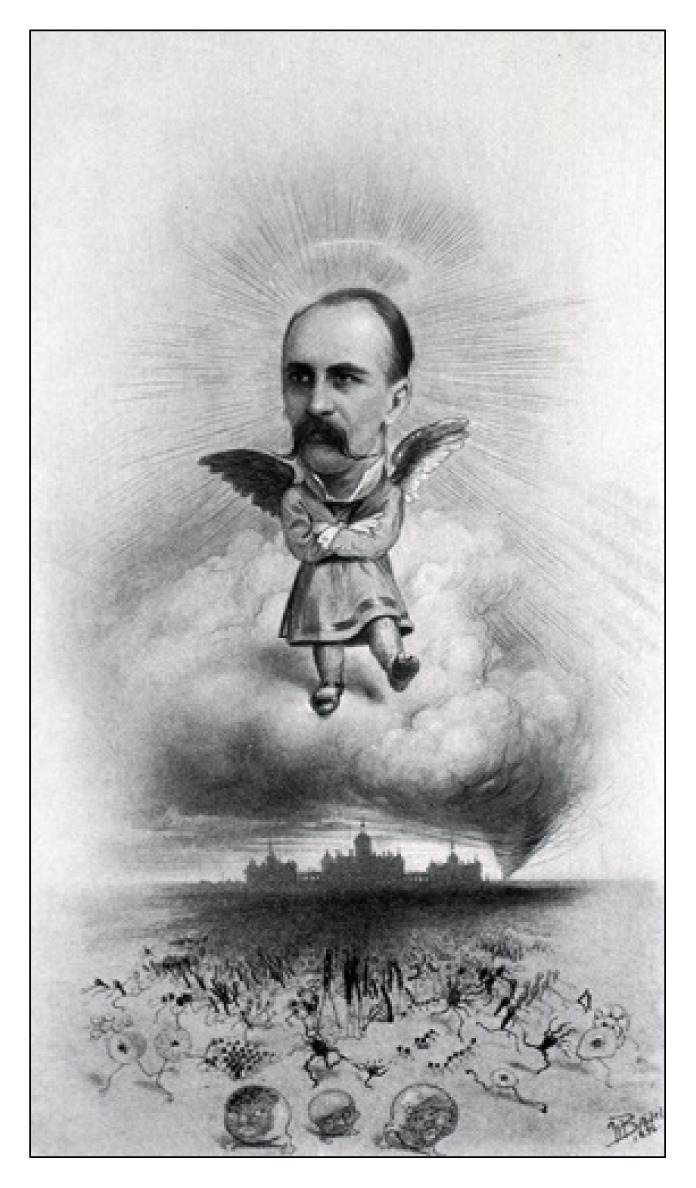


There are times when something is simply so familiar that we can no longer see it at all. It can be a story, or a concept, or even a flesh-and-blood person. Familiarity breeds not only contempt, but a kind of invisibility as well. For too many of us, such is the case with Sir William Osler. In his time (1849–1919), many considered him to be one of the greatest practitioners, teachers, and writers ever in the field of medicine. He was instrumental in the founding of the Medical Library Association (MLA) and was elected its second president. He was gracious and witty, and he could grow the kind of mustache that only a true English gentleman could carry off, even though he was Canadian by birth and spent much of his professional career in the United States.

Today, if he is thought about at all in our profession, it is more likely as a sort of punchline. Any old chestnut of a quote is likely to be attributed to Osler. He was, in actuality, a great writer of maxims—”pneumonia may well be called the friend of the aged,” “soap and water and common sense are the best disinfectants,” “a man must have faith in himself to be of any use in the world”—but he didn’t say quite everything [1]. There is also the half-remembered bit (and he really DID say this) that men after the age of sixty were useless and should step aside so that younger men could come forward [2]. He wrote that in 1905, when he was fifty-six; he was still hard at work when he died at age seventy, with no plans to retire.

Osler was a formidable bibliophile and lover of libraries. I have already mentioned his link to MLA, and tradition has it that he convinced Marcia Noyes to become a medical librarian, offering her what instruction she needed in the field [3]. But this was all a very long time ago, and it is fair to ask: does Sir William still matter to us, as individuals, as a profession?

To answer that question, one must look to his writings; not to his textbooks (although it is worth noting that his 1,100-page *Principles and Practices of Medicine,* first published in 1892 and a standard text for decades, was essentially the work of just Osler), but to his essays and speeches. The most famous collection is *Aequanimitas,* published in 2 editions in Osler’s lifetime (1904 and 1906) and further expanded after his death. Most of the chapters are speeches and formal addresses in a style that is both quaint and forbidding to the modern reader, full of taglines from classical authors and Victorian poets, and with a definite aura of port and cigars in an exclusive gentleman’s club. One paragraph into the title piece, and we have Plato, Marcus Aurelius, and Matthew Arnold, to say nothing of the whopping title itself: the last word of a dying Roman emperor.

It is time to give Osler a bit of slack. After all, he delivered “Aequanimitas” to the faculty of the University of Pennsylvania in May of 1889 as he left for the brand-new Johns Hopkins, and Matthew Arnold had died barely a year before. Osler was about to enter the most famous and fruitful phase of his career, and he knew it. He can be granted a bit of (somewhat tendentious) reflection, especially since pompous speeches were expected of any such man in similar circumstances. But we are permitted to inquire what “Aequanimitas” is about.

The answers is deceptively simple. Osler is talking about imperturbability—the first cousin of Hemingway’s definition of guts: grace under pressure. Osler expects that a physician be forever calm and even distant from the tumult that may surround the patient’s situation. He suggests that “a certain measure of insensitivity is not only an advantage, but a positive necessity in the exercise of calm judgement.” What might Osler have made of Mr. Spock from *Star Trek?* And do we want either Osler or Spock as our physician?

Further on in the collection (which is largely, but not entirely, chronological), Osler grows more mellow. In his talk on “Books and Men,” Osler muses on the role of libraries, and especially medical libraries, in his early life. In 1876, as a young professor at McGill University, he first visited the Boston Medical Library, after finding that his own institution lacked the library resources that he needed for his work. “It was a small matter,” he writes, but it is also over 300 miles from Montreal to Boston. He found the references he needed, but he also found “a cordial welcome and many friends.” We are allowed to wonder which was the more important, but it strikes me that it might well be the latter. Osler was a prodigious networker, and his eventual network was very large indeed.

This is not to say that Osler’s high regard for libraries was anything but deeply felt and obviously sincere. While he spent his career in academic settings with many books and esteemed colleagues, he also considered the travails of the solitary practitioner. A library, he deemed, was essential if the lonesome doc was to survive, both emotionally and professionally.

However, it is in the address “Some Aspects of American Medical Bibliography” that Osler comes closest to us in the present day. Delivered in 1902, it was his inaugural address as president of the Association of Medical Librarians—MLA’s original name. As mentioned earlier, he was the second president of our association, succeeding the founding president, George M. Gould. Gould could be spikey and overly zealous; he would eventually antagonize many in the association that he helped to found. Osler, urbane and conciliatory, proved to be the better model for a president (although it would be many years before an actual librarian would be elected to the post). [4]

As one would expect, Osler starts from a very pragmatic place. He speaks of big libraries and small ones, how they interact and what they owe to each other. This leads him to a discussion of the Exchange, the clearinghouse for the transfer of duplicates and such that was MLA’s major activity for many decades. But he is more interested in the bibliography of medicine, in the grand sense of that work. There are gems, he says, and they need to be polished.

And that is, I believe, his most urgent message for the contemporary librarian (and archivist!). We are the memory of our field. We must remember where we came from, if we are to make sense of where we are going. Gem polishing can take many forms (both digitizing and conserving will qualify). I think there is a middle way that would make Sir William glad. That is why it is so important, as a profession, to remember.

Individuals who are interested in writing for “History Matters” should contact Stephen Greenberg, MSLS, PhD, greenbes@mail.nih.gov, Section Head, Rare Books and Early Manuscripts, History of Medicine Division, National Library of Medicine, 8600 Rockville Pike, Bethesda, MD 20894.

This work was supported by the Intramural Research Program of the National Institutes of Health, National Library of Medicine.
